# Proof-of-Concept of a Sensor-Based Evaluation Method for Better Sensitivity of Upper-Extremity Motor Function Assessment

**DOI:** 10.3390/s21175926

**Published:** 2021-09-03

**Authors:** Seung-Hee Lee, Ye-Ji Hwang, Hwang-Jae Lee, Yun-Hee Kim, Matjaž Ogrinc, Etienne Burdet, Jong-Hyun Kim

**Affiliations:** 1 KEPCO Research Institute, Seoul 06732, Korea; s.lee@kepco.co.kr; 2 School of Mechanical Engineering, Sungkyunkwan University, Suwon 16419, Korea; yj.hwang@skku.edu; 3 Center for Prevention & Rehabilitation, Heart Vascular and Stroke, Samsung Medical Center, Department of Physical and Rehabilitation Medicine, Sungkyunkwan University School of Medicine, Seoul 06351, Korea; goodptlee@skku.edu (H.-J.L.); yunkim@skku.edu (Y.-H.K.); 4 Department of Bioengineering, Imperial College London, London SW72AZ, UK; matjaz.ogrinc@imperial.ac.uk (M.O.); e.burdet@imperial.ac.uk (E.B.); 5 GripAble Limited, Thornton House, 39 Thornton Road, London, SW19 4NQ, UK

**Keywords:** sensor-based motor function assessment, depth sensor, fuzzy inference system, sensitivity, rehabilitation

## Abstract

In rehabilitation, the Fugl–Meyer assessment (FMA) is a typical clinical instrument to assess upper-extremity motor function of stroke patients, but it cannot measure fine changes of motor function (both in recovery and deterioration) due to its limited sensitivity. This paper introduces a sensor-based automated FMA system that addresses this limitation with a continuous rating algorithm. The system consists of a depth sensor (Kinect V2) and an algorithm to rate the continuous FM scale based on fuzzy inference. Using a binary logic based classification method developed from a linguistic scoring guideline of FMA, we designed fuzzy input/output variables, fuzzy rules, membership functions, and a defuzzification method for several representative FMA tests. A pilot trial with nine stroke patients was performed to test the feasibility of the proposed approach. The continuous FM scale from the proposed algorithm exhibited a high correlation with the clinician rated scores and the results showed the possibility of more sensitive upper-extremity motor function assessment.

## 1. Introduction

In rehabilitation, upper-extremity motor function evaluation for stroke survivors is important to plan effective rehabilitation intervention [1,2]. The most widely used in-person assessment in clinics is the Fugl–Meyer assessment (FMA) due to its validity and reliability [3,4,5]. Despite its popularity, FMA is (1) labor-intensive and time-consuming, and (2) not sensitive enough to fine changes in motor function ability due to the coarse three point grading scheme of the FM scale [4]. Although this grading scheme results in high inter/intra-rater reliability, it also has lower sensitivity than other clinical instruments, such as the medical research council muscle strength scale (six point scale) [4,5,6]. Many clinical studies have reported this limitation, meaning that it is not possible to track fine changes of a patient’s motor function using the FM scale [4,7,8,9,10].

Thanks to recent advances in sensor technologies, several works had reported an automated FMA system to address labor-intensiveness and time consumption issues [11], these, however, did not attempt to propose a more sensitive FM scale using the virtue of sensor-based measurements to overcome the limitation on low sensitivity [12,13,14,15,16,17,18]. It might be because most work to automate FMA showed inadequate accuracy even though the work focused on predicting the original three point FM scale. Another reason would be that the machine learning methods used in the existing works for FMA are not appropriate to handle this issue because (1) some of them (support vector machine [17,19] and Naive–Bayes classification [20]) cannot be used for regression and (2) the others (extreme machine learning [14], artificial neural network [15], and random forest [21,22]) require a large amount of dimension-reduced training data for regression, which could not be collected from numerous patients in practice.

A promising solution for a more sensitive FMA is to develop a continuous scoring algorithm for sensor-based automated FMA. Meanwhile, from our first attempt to apply a sensor-enabled body tracking to the FMA automation [17], we recently reported a sensor-based automated FMA system with a rule-based expert with binary logics originated from the linguistic grading guideline of FMA, which is different to the machine learning methods used in the other existing works [13]. Importantly, the binary logic was verified by high grading accuracy with the original FM scale [13] and could be applied to continuous scoring through the fuzzy logic approach, such as designing an appropriate fuzzy inference system (FIS) [23,24]. This FIS-based approach is promising because it (1) does not require collecting a large amount of patients’ data, (2) can consider a clinician’s ambiguous judgement mathematically [24,25] and (3) makes its reasoning process understandable [24].

The goal of this study was to check the feasibility of sensor-based continuous FM scale scoring. For that, we firstly chose three representative FMA tests and developed a novel scoring algorithm for the tests based on FIS defining the fuzzy variables and rules from the FMA guideline [23,24,25,26]. Then, a sensor-based automated FMA system that can provide continuous FM scale was implemented by using the scoring algorithm and a depth sensor (Kinect V2). After investigating the achievable number of grades under the system by considering the expected error of the sensor, we showed the feasibility of the proposed scoring method through a pilot trial with nine stroke patients.

## 2. Materials and Methods

### 2.1. Target FMA Tests and Sensor Selection

As the targets for developing a continuous FM scale scoring algorithm, we selected three tests out of the FMA tests automated in our previous study [13], which are listed in Table 1. These tests consist of volitional movements with synergies and without synergy [3], and all of them have quite different binary logics to prove the feasibility of the proposed approach [13].

### 2.2. Continuous FM Scale Scoring Algorithm

#### 2.2.1. Fuzzy Variables

Based on the features used in our FMA studies [13,17], we determined the fuzzy input variables (***X***) for each target test as shown in Table 2. The fuzzy input variables can be divided into three types: *F*_Va_ and *F*_Vb_ for evaluating the selective/voluntary motor performance in three and two stages, respectively, and FM for evaluating the ability to maintain a specific constraint (posture) in two stages (Table 2). Therefore, the fuzzy set (***R***) of the fuzzy input variables (***X***) can be summarized as shown in Table 2. Note that we used the same feature extraction method [13] to obtain the variables.

The fuzzy output variables (***Y***) are the FM scores for the target tests. Each FM score was assigned three levels (0: cannot be performed at all; 1: can be performed partially; and 2: can be performed fully) according to the degree of motor function judged by the clinician. This means that the fuzzy sets (***S***) of the fuzzy output variables were classified as shown in Table 3.

#### 2.2.2. Fuzzy Rules

The propositions expressed in words can be transformed into fuzzy rules for the scoring FM scale by using the explicitation process [24,25]. All established fuzzy rules can be defined as the following fuzzy canonical form:

(1) IF X is R THEN Y is S

From the viewpoint of the FMA, ***X*** is the clinician’s observation, ***R*** is the clinician’s judgment regarding the performance level of each feature, ***Y*** is the degree of the patient’s motor function, and ***S*** is the FM scale assigned by the clinician.

The fuzzy rules of the target tests are summarized in Table 4. T1 had the simplest logical structure with three rules; an *F*_Va_ is evaluated at three performance levels without maintaining a specific posture. As for T2, two rules were added to the rules of T1 in order to consider the ability of posture constraint. T3 consisted of three rules, which were to evaluate two *F*_Vb_ in two levels without posture constraint. It should be noted that the weights among all fuzzy rules were set to 1.

#### 2.2.3. Fuzzy Inference System

We adopted the Mamdani method to implement FIS. This method has the characteristic that inference results can be easily transformed into linguistic forms [25].

The fuzzy set ***R*** of ***X*** in (1) can be defined as follows:

(2) $$R=\left\{\left(x,\;\mu_{R}\left(x\right)\right):x\in X,\;\mu_{R}\left(x\right)\in \left[0,\;1\right]\right\}\;\mathrm{with}\;X:\;\alpha \leq x\leq \beta ;\;\mu_{R}:X\to \left[0,\;1\right]$$

where x denotes the feature measured by the sensor; $\mu_{R}$ the membership function (MF) for the fuzzification of ***X***; and *α* and *β* are the minimum and maximum values within the universe of discourse, respectively. Here, *α* was set to 0, and *β* was determined as the desired feature value in the instructed motion or as 30 for the SD features (*F*_M_6_ to *F*_M_9_) [13].

Like (2), the fuzzy set ***S*** of ***Y*** in (1) was defined as shown below:

(3) $$S=\left\{\left(y,\;\mu_{S}\left(y\right)\right):y\in Y,\;\mu_{S}\left(y\right)\in \left[0,\;1\right]\right\}\;\mathrm{with}\;Y:\;\gamma \leq x\leq \delta ;\;\mu_{S}:Y\to \left[0,\;1\right]$$

where *y* denotes the degree of a patient’s motor function judged by a clinician; $\mu_{S}$ is the MF for the implication of ***Y***; *γ* and *δ* were 0 and 1, respectively.

**Figure 1:**
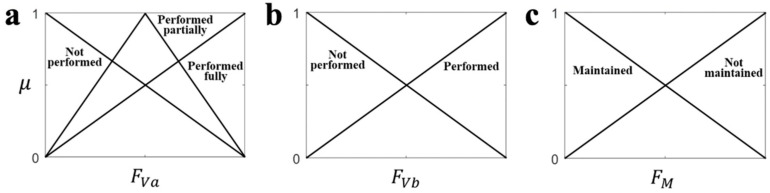
The membership functions for each type of feature. (**a**) *F*_Va_, (**b**) *F*_Vb_, (**c**) *F*_M_.

For the MFs in (2) and (3), we adopted a triangular shaped function that is simple and widely used for modeling the human’s reasoning process [27,28,29], as displayed in Figure 1. The MF in the fuzzy set *R*_1_ for *F*_Va_ was designed to satisfy the fact that a clinician’s judgment approaches ‘performed fully’ when the measured feature value is closer to the desired value in the instructed motion (Table 2). The opposite meaning, ‘not performed’, was implemented as the MF in *R*_3_ using the complement of *R*_1_ (Figure 1). We also designed the MF in *R*_2_ with the assumption that the clinician’s ‘performed partially’ judgement results in the highest degree of membership when the measured feature value was half of the desired feature value (Figure 1). The MFs of the fuzzy sets ***S*** for ***Y*** were also designed with the same shape. Moreover, for *F*_Vb_ and *F*_M_, which have two stages in FMA, the MFs in *R*_4_ (*R*_6_) and *R*_5_ (*R*_7_) were designed as the same shape to the MFs of *R*_1_ and *R*_3_, respectively.

**Figure 2:**
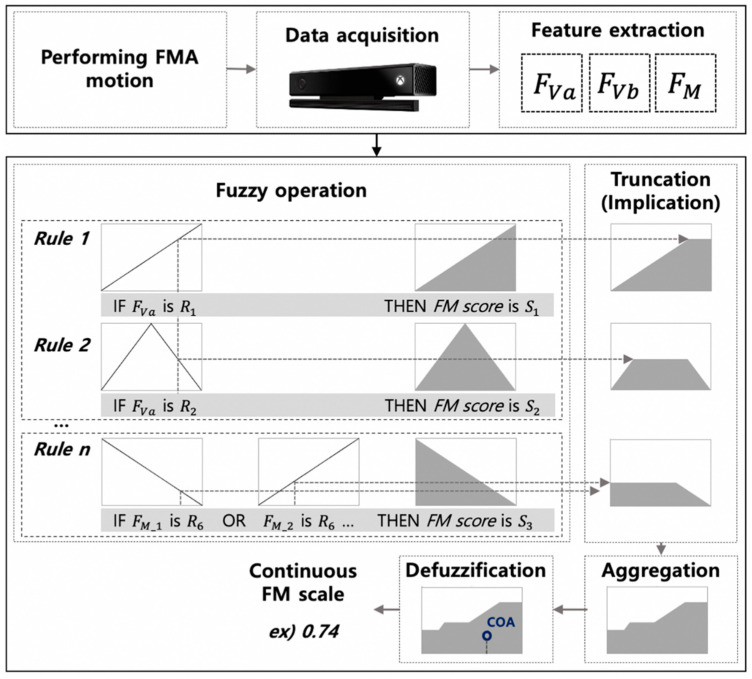
Schematic diagram of the continuous FM scale scoring algorithm; COA denotes the center of area in the centroid defuzzification method [31].

The logic rules AND/OR were formulated by the minimum/maximum functions. The AND operation was applied for the truncate (implication) of ***S***, and the maximum function was used for aggregation operation [30]. The result of the designed FIS, continuous FM scale, was provided as a constant value by using centroid defuzzification process [30,31]. The continuous FM scale scoring algorithm through FIS using the data acquired by the sensor is represented as shown in Figure 2.

In summary, based on the rule-based logic obtained from the linguistic grading guidelines of FMA [13], we have developed a novel continuous FM scale scoring algorithm using a fuzzy logic approach. In contrast to a machine learning approach, our algorithm can easily rate a continuous FM scale without requiring any training data including a clinician’s three point FM score.

#### 2.2.4. The Sensor-Based Automated FMA System’s Achievable Number of Grades

We implemented a sensor-based automated FMA system. Similar to our previous study [13], the system consisted of a sensor, user interface, and scoring algorithm. A depth sensor, Kinect V2 (Microsoft, Redmond, WA, USA) was used to extract motion features (Figure 2), which is an inexpensive and easy to use sensor that has been widely applied in the rehabilitation area [13,15,16,17,32], and whose effectiveness was verified in our previous FMA studies [13,17]. The system has the same user interface as our previous automated FMA system [13], which provided an instruction video that was prerecorded by a well-experienced clinician. In contrast to our previous system, the system in this study could provide a continuous FM scale due to the proposed continuous FM scale scoring algorithm.

The continuous FM scale due to the scoring algorithm (FM_CA_), which comes from the fuzzy result value of the FIS, would be a solution for better sensitivity of the FMA. However, the inaccurate input value due to measurement error of the depth sensor could restrict the achievable number of FM grades. To investigate this restriction, the confidence range of fuzzy result value was analyzed as follows. After simulating the expected maximum (worst) feature errors by using the reported tracking error of the depth sensor used (Kinect V2) [33] considering the effect of sensor position [34] and anthropometric data [35] (Table 5), we estimated the possible maximum fuzzy result errors and number of grades, as represented in Table 6. The results showed that FM_CA_ could be interpreted up to seven scale in all the target FMA tests (Table 6), which implies that motor function could be more sensitively evaluated than the conventional three point FM scale.

### 2.3. Experiment

#### 2.3.1. Experimental Setup

**Figure 3:**
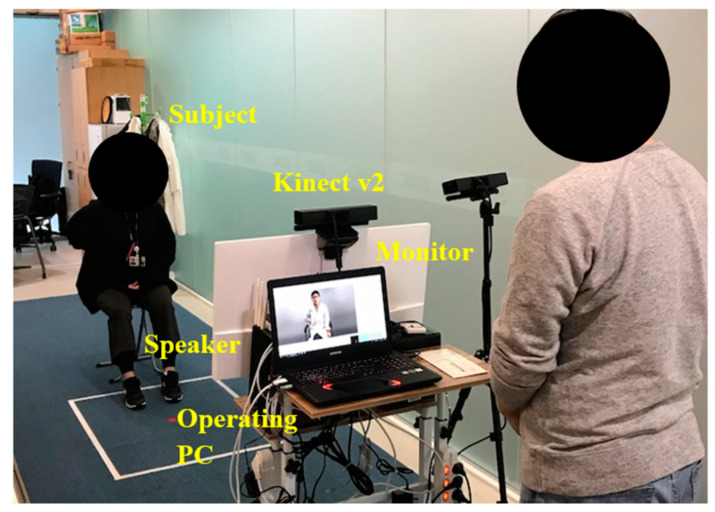
Experimental setup. Note that another Kinect that is located beside the monitor was just used to record depth images during FMA tests.

We conducted an experiment using the implemented system with stroke patients to test the feasibility of the proposed continuous FM scale scoring algorithm. Figure 3 shows the experimental setup. A Kinect V2 sensor was installed one meter in front of the subject (Figure 3), and data were recorded at a sampling rate of 30 Hz. The instructions were delivered to the subject through the instruction video. For the purpose of showing the video, a monitor (visual) and speaker (auditory) were installed near the sensor (Figure 3). The sensor data were automatically recorded after the start of the video, and this recording was finished when the subject’s movement was completed.

#### 2.3.2. Protocol

Nine stroke patients (ages 49 to 77 years) participated in the experiment, whose biographical information is summarized in Table 7. This experiment was approved by the Samsung Medical Center institutional review board (SMC-2018-02-053), and all subjects gave their consent prior to the experiment.

The subjects sat in a chair without an armrest or wheelchair (Figure 3). They were asked to follow the motions in the instruction video, and thus they mimicked the motion while the video was being played. During the subjects’ motion, a well-experienced (more than 10 years) clinician observed the motion, and rated the conventional three point FM scale (FM_3_) as well as the following extended seven point FM scale (FM_7_):

**0**: cannot be performed at all (same to FM_3_);
**0+**: can be performed a little bit but close to level that cannot be performed at all;
**1−**: can be performed partially but close to level that cannot be performed;
**1**: can be performed partially (same to FM_3_);
**1+**: can be performed partially but close to level that can be performed well;
**2−**: can be performed well but not perfectly;
**2**: can be performed perfectly (same to FM_3_).

The reason why we used the FM_7_ above was that it would not be enough to evaluate the proposed FM_CA_ using FM_3_, considering the achievable number of grades estimated. In order to reduce the clinician’s scoring difficulty, FM_7_ was made as a straightforward extension by adding ‘0+’, ‘1−’, ‘1+’ and ‘2−’ to the FM_3_.

#### 2.3.3. Data Analysis

Sensor data of 27 trials (three target FMA tests with nine patients) were recorded during the experiment. The high frequency spikes and jitters in the data were removed through a third-order low-pass Butterworth filter (10 Hz cutoff frequency) [16]. Then, two different FM scales were obtained from the data: (1) a three point FM scale using the automated FMA algorithm in our previous study (FM_3A_) [13], and (2) FM_CA_ (continuous FM scale due to the proposed FIS-based scoring algorithm) (Figure 2). Here, the FIS was implemented by using a fuzzy logic toolbox in Matlab (Mathworks, Natick, MA, USA), and the fuzzy result value (FM_CA_) was linearly normalized to have the range from 0 to 1.

In order to validate the quality of the collected data in this experiment, we investigated whether the data could provide an accurate three point FM scale. The agreement and Cohen’s kappa were calculated between the FM_3A_ and FM_3_. From this analysis, one can indirectly deduce whether the inaccurate FM_CA_ is due to low data quality or erroneous fuzzy rule/FIS design.

The proposed FM_CA_ needs to have the following clinical characteristics: FM_CA_ corresponds to the patients’ degree of motor function evaluated by a clinician. For that, we calculated the Pearson’s correlation coefficient between the FM_CA_ and FM_3_/FM_7_ [30]. Moreover, in clinic, since FMA used the total sum of the FM scale for each FMA test to evaluate a patient’s overall motor function [4], the Pearson’s correlation coefficient between the sum of FM_CA_ and sum of FM_3_/FM_7_ was calculated to check whether the proposed FM_CA_ could also follow the overall evaluation method [13,14,15,16]. The statistical analysis was performed using SPSS version 20 (IBM, Chicago, IL, USA).

## 3. Results

All the subjects’ FM scales were automatically rated with three point level (FM_3A_) and continuous level (FM_CA_) by using our automated FMA system developed in [8], along with the manually scored FM_3_ and FM_7_ as summarized in Table 8.

### 3.1. Validity of Collected Data

The agreement between FM_3A_ and FM_3_ was 96.3%, and Cohen’s kappa was 0.940, as summarized in Table 9. This result was similar to our previous result for the automated FMA system (92% agreement; 0.877 Cohen’s kappa) [13]. It supports the validity of the quality of data collected in this study. Disagreement only occurred in a T3 trial in which the clinician rated score ‘2’ (Table 9).

### 3.2. Continuous FM Scale

**Figure 4:**
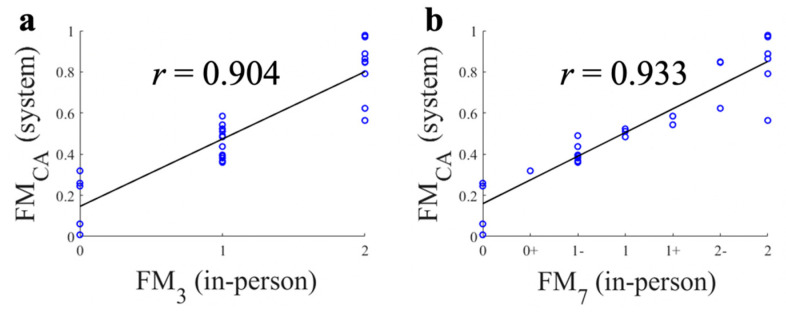
Correlations between continuous FM scale (FM_CA_), three point FM scale (FM_3_), and seven point FM scale (FM_7_). (**a**) FM_CA_ and FM_3_; (**b**) FM_CA_ and FM_7_.

Figure 4 shows the correlations between FM_CA_ and FM_3_/FM_7_ in the 30 trials. Overall, a high Pearson’s correlation coefficient was observed for FM_3_ (*r* = 0.904) and FM_7_ (*r* = 0.933) (Figure 4), and it was also valid for each FMA test (T1: *r* = 0.930 for FM_3_, *r* = 0.959 for FM_7_, T2: *r* = 0.897; for FM_3_, *r* = 0.966; for FM_7_; and T3: *r* = 0.896; for FM_3_, *r* = 0.903; for FM_7_). These results showed that the proposed FM_CA_ corresponded to the FM_7_ rated by the clinician.

**Figure 5:**
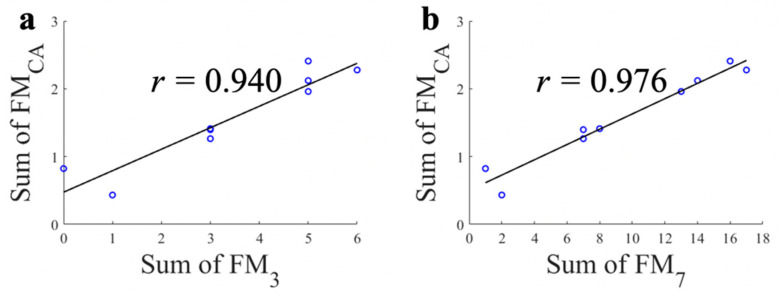
Correlations between the total sum of each FM scale. (**a**) Proposed continuous FM scale (FM_CA_) and conventional three point FM scale (FM_3_); (**b**) Proposed continuous FM scale (FM_CA_) and extended seven point FM scale (FM_7_).

The correlations between the total sum of FM_CA_ and FM_3_/FM_7_ for each subject are shown in Figure 5. Note that there was no subject who had a total sum of FM_3_ as two or four (Figure 5a). The high correlation results (*r* = 0.940; with FM_3_ and *r* = 0.976; with FM_7_) show that the proposed FM_CA_ system can estimate overall motor function accurately (Figure 5). Here, the total sum of FM_7_ in Figure 5b was calculated through the conversion of FM_7_ as ‘1−’ to ‘2’, ‘1’ to ‘3’, ‘1+’ to ‘4’, ‘2−’ to ‘5’ and ‘2’ to ‘6’. It should be note that all correlation analyses above showed significant correlation (*p* < 0.001).

## 4. Discussion

In this study, we used FM_7_ along with FM_3_ to evaluate the proposed FM_CA_. The clinician reported that the rating of FM_7_ was not difficult because FM_7_ is a simple scale expansion of FM_3_. The extended scales in FM_7_ (0+, 1−, 1+, and 2−) appeared in 51.9% of the total FMA tests (14 out of 27). This means that there is a clear demand in clinic for evaluating motor function by using a more sensitive FM scale than the existing FM_3_. It should be noted that FM_7_ could not currently be regarded as a validated clinical tool.

The T3 FMA test resulted in lower correlation (*r* = 0.903) than T1 and T2, because of a disagreement in a trial between FM_3A_ and FM_3_ highlighted in Table 8. The correlation becomes much higher (*r* = 0.984) when this trial is excluded. Since the FM_7_ of the trial were ‘2’, FM_CA_ had the greatest deviation for trials that belong to score ‘2’ (Figure 4). We believe that the lower performance in T3 was caused by inaccurate tracking of the motion sensor used (Kinect V2). For T3, we extracted two *F*_Vb_ features when the subject moved the hand to the knee. Here, one of the features, shoulder inward rotation ROM, could not be precisely extracted because when the subject’s distal segment of the upper limb was moving along the proximal direction, the subject’s loose patient uniform, made the measurement of the angle unreliable (about a 16 degree error) [13]. If the proposed system was applied to 26 FMA tests, we expect that 22 of 26 tests would be free from the sensor inaccuracy problem above based on the characteristics of the inaccurate tracking investigated in [13], except the following tests: shoulder adduction/inward rotation during hand to knee (T3), shoulder external rotation during hand to ear, forearm supination during hand to ear, and forearm pronation/supination with elbow 0°.

As mentioned, this paper proposed a novel continuous FM_CA_ scoring algorithm based on the fuzzy logic derived from our previous rule-based expert (binary logics). One can expect that several existing studies on automated FMA could be extended for the continuous FM scale. For instance, a linearized model that is obtained from the correlation analysis between the extracted feature (i.e., range of motion) and original FM scale rated by clinician could enable the scoring of the continuous scale [16]. Those approaches, however, would suffer from inaccuracies due to the complexity of FMA (i.e., Pearson’s correlation coefficient *r* = 0.03 in some tests [16]), as follows. Based on the Bobath concept [3,4], the instructions of FMA usually ask the patient to perform a certain joint motion while constraining the other joint motions for evaluating the selective/voluntary motor performance. Hence, the FM scale is rated by clinician’s comprehensive inference based on multiple features with different types: *F*_Va_, *F*_Vb_, and *F*_M_, and thus it makes the dimension reduction used in those approaches (i.e., using principal component analysis [16]) difficult. Note that this statement is supported by the complex binary logic for automating some FMA tests that were shown in our previous work [13].

The aim of the proposed sensor-based continuous-scaled FMA system is to automate the evaluation of motor function more objectively and sensitively. From a clinical point of view, along with its convenience and time efficiency, the proposed system has the potential to improve the limited sensitivity of the conventional FM scale, which would be a novel instrument for better practice of rehabilitation. Moreover, the proposed system can contribute to effective robot-aided rehabilitation therapy due to its better sensitivity. For instance, thanks to FM_CA_, the intensity and difficulty of the robotic therapy can be precisely chosen, and the fine monitoring of the motor function after the therapy could be used to accurately investigate its therapeutic effect. In addition, the proposed system is promising to be utilized as a key measure for achieving precise big data for upper-extremity motor function.

This study could still be improved. We only implemented three FMA tests for the proposed continuous FM scale so as to investigate its feasibility. Since the rule-based binary logic, the basis of FIS, for most FMA tests was already found in our previous work [13], it is promising that the unimplemented tests could be covered in a similar manner in the near future. As for the sensor system, the performance could be improved when we use a state-of-art depth sensor, such as RealSense (Intel, Santa Clara, CA, USA) or Leap motion controller (Leap Motion Inc., San Francisco, CA, USA) [36], both of which have better resolution than Kinect only. Moreover, the reliability (consistency) test of the proposed FMCA with repeated trials and various environment would be needed to confirm the feasibility of the proposed approach. In addition, the limited number of subjects in this study could be solved through an additional clinical trial with a larger population.

## 5. Conclusions

FMA, a well-known clinical instrument for stroke patients, still has low sensitivity, so it cannot evaluate fine changes of motor function. As a solution to this limitation, this study showed the possibility that sensor-based automated FMA system with the proposed FIS-based algorithm could provide a continuous FM scale (FM_CA_), which is highly correlated with the conventional FM scale (FM_3_) as well as the extended FM scale (FM_7_). It means that the designed FIS in the system for scoring FMCA faithfully reflects the clinical knowledge of FMA. This is additionally supported by the high correlations between the total sum of FM_CA_ and FM_3_/FM_7_. To our knowledge, this study is the first attempt (1) to develop the continuous FM scale and (2) to apply fuzzy logic approach (i.e., FIS) for automated and more sensitive FMA. Therefore, we expect that the proposed system could be a basis to improve the quality of motor function assessment for stroke patients significantly.

## Figures and Tables

**Table 1:** Target FMA tests.

FMA Test	Index
Elbow extension during hand to knee	T1
Shoulder abduction 0–90°	T2
Shoulder adduction/inward rotation during hand to knee	T3

**Table 2:** Fuzzy input variables and their fuzzy sets.

**Fuzzy Input Variables (*X)***			
**FMA Test**	**Type**	**Index**	**Description**
T1	*F*_Va_	*F*_Va_	Elbow extension ROM
T2	*F*_Va_	*F*_Va_	Shoulder abduction ROM
	*F*_M_	*F*_M_1_	Shoulder abduction angle (mean, onset)
		*F*_M_2_	Elbow flexion angle (mean, onset)
		*F*_M_3_	Shoulder flexion angle (mean, onset)
		*F*_M_4_	Elbow flexion angle (mean, motion)
		*F*_M_5_	Forearm pronation angle (mean, motion)
		*F*_M_6_	Elbow flexion (SD, onset)
		*F*_M_7_	Shoulder flexion angle (SD, onset)
		*F*_M_8_	Elbow flexion angle (SD, motion)
		*F*_M_9_	Forearm pronation angle (SD, motion)
T3	*F*_Vb_	*F*_Vb_1_	Shoulder abduction ROM
		*F*_Vb_2_	Shoulder inward rotation ROM
**Input Fuzzy Sets (*R)***			
**Type**	**Index**	**Description**	
*F*_Va_	*R*_1_	Performed fully	
	*R*_2_	Performed partially	
	*R*_3_	Not performed	
*F*_Vb_	*R*_4_	Performed	
	*R*_5_	Not performed	
*F*_M_	*R*_6_	Maintained	
	*R*_7_	Not maintained	

ROM denotes the range of motion; SD the standard deviation. ‘Onset’ and ‘motion’ denote the onset phase and the motion phase, respectively.

**Table 3:** Fuzzy output variables and their fuzzy sets.

Fuzzy Output Variables (*Y*)	Output Fuzzy Sets (*S*)	
	Index	Description
FM score	*S*_1_	High motor function as FM score ‘2’
	*S*_2_	Medium motor function as FM score ‘1’
	*S*_3_	Low motor function as FM score ‘0’

**Table 4:** Fuzzy rules of each target FMA test.

FMA Test	# of Rules	Description
T1	3	(1) IF *F*_Va_ is *R*_1_ THEN *FM score* is *S*_1_ (2) IF *F*_Va_ is *R*_2_ THEN *FM score* is *S*_2_ (3) IF *F*_Va_ is *R*_3_ THEN *FM score* is *S*_3_
T2	5	(1, 2, 3) same as T1 fuzzy rules (4) IF *F*_M_1_ is *R*_7_ OR *F*_M_2_ is *R*_7_ OR *F*_M_3_ is *R*_7_ OR *F*_M_6_ is *R*_7_ THEN *FM score* is *S*_3_ (5) IF *F*_M_4_ is *R*_7_ OR *F*_M_5_ is *R*_7_ OR *F*_M_8_ is *R*_8_ OR *F*_M_9_ is *R*_7_ THEN *FM score* is NOT *S*_1_
T3	3	(1) IF *F*_Vb_1_ is *R*_4_ AND *F*_Vb_2_ is *R*_4_ THEN *FM score* is *S*_1_ (2) IF *F*_Vb_1_ is *R*_5_ OR *F*_Vb_2_ is *R*_5_ THEN *FM score* is *S*_2_ (3) IF *F*_Vb_1_ is *R*_5_ AND *F*_Vb_2_ is *R*_5_ THEN *FM score* is *S*_3_

**Table 5:** Information used to estimate fuzzy result error due to sensor inaccuracy.

**Joint Tracking Error of Kinect V2**		
**Kinect V2 Landmark**	**Mean Error (Standard Deviation)**	**FMA Tests Related**
Shoulder	1 cm (1 cm)	T1, T2, T3
Elbow	2 cm (1 cm)	T1, T2, T3
Wrist	2 cm (1 cm)	T1
Spine shoulder	1 cm (1 cm)	T2, T3
Spine mid	1 cm (1 cm)	T2, T3
Shoulder internal rotation	16 deg	T3
**Used Anthropometry**		
**Body segment**	**Length**	**FMA tests related**
Upper arm (shoulder to elbow)	27.2 cm	T1, T2, T3
Lower arm (elbow to wrist)	29.4 cm	T1
Torso	90.1 cm	T2, T3

Note that anthropometry data were from a 40 year old Asian female [35].

**Table 6:** Estimated fuzzy result error and possible number of grades for each FMA test.

Target FMA Tests	Feature	Maximum Feature Error (Deg)	Maximum Fuzzy Result Error	Possible # of Grades
T1	*F*_Va_	23.382	0.104	9
T2	*F*_Va_	14.432	0.128	7
	*F*_M_1_	14.432		
	*F*_M_2_	23.382		
	*F*_M_3_	14.432		
	*F*_M_4_	23.382		
	*F*_M_5_	7.81		
	*F*_M_6_	6.222		
	*F*_M_7_	2.934		
	*F*_M_8_	6.222		
	*F*_M_9_	4.52		
T3	*F*_Vb_1_	14.432	0.081	12
	*F*_Vb_2_	16		

Note that the features for each test can be found in Table 2.

**Table 7:** Biographical information of participated stroke patients.

	Age/Sex	Affected Side	Time Since Stroke (Month)	Etiology	MMSE	UE–FMA
S1	49/F	Left	15	Rt. FT ICH	30	12
S2	54/M	Left	18	Rt. LS inf.	28	27
S3	57/M	Right	32	Lt. Pons inf.	29	62
S4	69/F	Right	3	Lt. Pons inf.	27	63
S5	58/F	Right	50	Lt. BG ICH	25	23
S6	61/M	Left	1	Rt. MCA inf.	29	37
S7	77/F	Left	3	Rt. Thalamus ICH	24	9
S8	65/M	Left	1	Rt. Thalamus ICH	28	12
S9	68/M	Right	1	Lt. MCA territory	27	8

F and M denotes female and male, respectively; MMSE denotes Mini Mental State Examination score; UE–FMA is the total sum of conventional three point FM scale (FM_3_). Rt = Right; Lt = Left; FT = Frontotemporal; LS = Lenticulostriate; BG = Basal ganglia; MCA = Middle cerebral artery; F = Frontal; ICA = Internal cerebral artery; CR = Corona radiata; ICH = Intracerebral hemorrhage; inf. = Infarction.

**Table 8:** Rated FM scores by clinician and implemented sensor-based system.

	T1				T2				T3			
	FM_3_	FM_3A_	FM_7_	FM_CA_	FM_3_	FM_3A_	FM_7_	FM_CA_	FM_3_	FM_3A_	FM_7_	FM_CA_
S1	1	1	1−	0.490	1	1	1−	0.396	1	1	1	0.511
S2	1	1	1−	0.390	1	1	1+	0.585	1	1	1−	0.437
S3	2	2	2	0.864	2	2	2−	0.849	**2**	**1**	**2**	**0.564**
S4	1	1	1+	0.543	2	2	2	0.978	2	2	2	0.888
S5	2	2	2	0.791	1	1	1	0.484	2	2	2−	0.846
S6	2	2	2−	0.623	1	1	1−	0.367	2	2	2	0.970
S7	1	1	1−	0.360	1	1	1−	0.381	1	1	1	0.523
S8	0	0	0	0.061	1	1	1−	0.364	0	0	0	0.008
S9	0	0	0	0.245	0	0	0	0.259	0	0	0+	0.319

Note that the bold numbers mean the disagreement between FM_3_ and FM_3A_. FM_3_, FM_3A_, FM_7_, and FM_CA_ denote clinician rated three point scale, system rated three point scale, extended seven point scale, and the proposed continuous scale, respectively.

**Table 9:** Agreement of FM_3_ and FM_3A_.

		FM_3A_ (System)			
		0	1	2	Total
FM_3_ (in-person)	0	**5**	0	0	5
	1	0	**13**	0	13
	2	0	1	**8**	9
	Total	5	14	8	27

Cohen’s kappa = 0.940; 96.3% agreement; Note that the bold text represents the number of agreed FM trials. FM_3_ and FM_3A_ denote clinician rated three point FM scale and system rated three point FM scale, respectively.

## Data Availability

Data and materials can be made available upon request to the authors.
